# The Relationship Between Health Literacy, Knowledge, Fear, and COVID-19 Prevention Behavior in Different Age Groups: Cross-sectional Web-Based Study

**DOI:** 10.2196/41394

**Published:** 2023-04-17

**Authors:** Mami Ishizuka-Inoue, Kanako Shimoura, Momoko Nagai-Tanima, Tomoki Aoyama

**Affiliations:** 1 Department of Human Health Sciences Graduate School of Medicine Kyoto University Kyoto Japan

**Keywords:** infodemic, COVID-19, health literacy, fear of COVID-19, cross-sectional study, behavior, age group, misinformation, influence, prevention, disease

## Abstract

**Background:**

COVID-19 prevention behaviors have become part of our lives, and they have been reported to be associated with health literacy, knowledge, and fear. However, the COVID-19 pandemic may be characterized by different situations in each age group. Since the severity of the infection and the means of accessing information differ by age group, the relationship between health literacy, knowledge, and fear may differ. Thus, factors that promote preventive behavior may differ by age group. Clarifying the factors related to prevention behaviors by age may help us consider age-appropriate promotion.

**Objective:**

This study aims to examine the association between COVID-19 prevention behaviors and health literacy, COVID-19 knowledge, and fear of COVID-19 by age group.

**Methods:**

A cross-sectional study was conducted among 512 participants aged 20-69 years, recruited from a web-based sample from November 1 to November 5, 2021. A web-based self-administered questionnaire was used to obtain the participants’ characteristics, COVID-19 prevention behaviors, health literacy, COVID-19 knowledge, and fear of COVID-19. The Kruskal-Wallis rank sum test was used to compare the scores of each item for each age group. The relationships among COVID-19 prevention behaviors, health literacy, COVID-19 knowledge, and fear of COVID-19 were analyzed using the Spearman rank correlation analysis. Additionally, multiple regression analysis was conducted with COVID-19 prevention behaviors as dependent variables; health literacy, COVID-19 knowledge, and fear of COVID-19 as independent variables; and sex and age as adjustment variables.

**Results:**

For all participants, correlation and multiple regression analyses revealed that prevention behaviors were significantly related to health literacy, COVID-19 knowledge, and fear of COVID-19 (*P*<.001). Additionally, correlation analysis revealed that fear of COVID-19 was significantly negatively correlated with COVID-19 knowledge (*P*<.001). There was also a significant positive correlation between health literacy and COVID-19 knowledge (*P*<.001). Furthermore, analysis by age revealed that the factors associated with prevention behaviors differed by age group. In the age groups 20-29, 30-39, and 40-49 years, multiple factors, including health literacy, influenced COVID-19 prevention behaviors, whereas in the age groups 50-59 and 60-69 years, only fear of COVID-19 had an impact.

**Conclusions:**

The results of this study revealed that the factors associated with prevention behaviors differ by age. Age-specific approaches should be considered to prevent infection.

## Introduction

The COVID-19 pandemic is ongoing, and prevention behaviors are now a part of our lives. The World Health Organization advised the public to adopt prevention behaviors, such as face masks and hand hygiene, and to avoid the 3Cs (ie, closed spaces, crowded places, and close-contact settings) [1]. Every individual should adopt such prevention behaviors to end the COVID-19 pandemic.

Several previous studies on COVID-19 prevention behaviors have reported associations with fear of COVID-19, health literacy, and COVID-19 knowledge. For example, a study conducted in the United Kingdom showed that fear of COVID-19 was a predictor of positive changes in COVID-19 prevention behaviors [2]. Fear of COVID-19 was assessed using the Fear of COVID-19 Scale (FCV-19S) [3], and has been reported to be associated with stress and depression [4]. Additionally, several positive associations between health literacy and preventive behaviors have been reported. During the COVID-19 pandemic, a study conducted in Norway on individuals aged 16-19 years showed an association between health literacy and handwashing behavior [5]. A study conducted among health care workers in Vietnam reported that the higher the health literacy, the better the compliance with infection prevention and control procedures [6]. A study conducted in Japan also reported an association between health literacy and prevention behaviors [7]. Health literacy is an individual’s personal knowledge and competencies to access, understand, evaluate, and use information and services to promote and maintain their own health and well-being and that of those around them [8]. Since COVID-19 has caused infodemics as well as pandemics, health literacy may be important in choosing true information and adopting correct behaviors [9]. In addition, those with more COVID-19 knowledge were reported to be more likely to engage in behaviors such as handwashing, avoiding enclosed spaces, and using public transportation and shopping less frequently, which suggests that COVID-19 knowledge is used in prevention behaviors [10,11]. In addition, fear, health literacy, and COVID-19 knowledge have also been reported to be interrelated. Previous studies have reported that higher health literacy leads to greater COVID-19 knowledge and that health literacy and COVID-19 knowledge reduce fear of COVID-19 [12-14]. All 3 factors are thought to interrelate and move people toward prevention behaviors.

However, COVID-19 pandemics are likely characterized by different situations in different age groups. COVID-19 is more likely to be severe in older people, while younger people are often asymptomatic or mildly ill [15]. Therefore, older people may be more fearful of infection than the younger population. In fact, a previous study has shown that the older one is, the greater the fear of COVID-19 [16]. Furthermore, in COVID-19 infodemics, misinformation about COVID-19 has been reported to have spread mainly through social network services (SNS) [17]. It has been reported that a high percentage of younger people receive information from SNS [18] and that the use of SNS may lead to misinformation [19,20]. Furthermore, health literacy has been reported to be lower in younger people [21]. Thus, younger people may be more likely to obtain and believe in misinformation than older people. As noted above, due to the different circumstances of different ages, the factors associated with prevention behavior may differ by age. Identifying this may help governments and local authorities tailor their approaches to promoting prevention behaviors to the characteristics of different age groups. In Japan, the Ministry of Health, Labour and Welfare is working to end the pandemic by publicizing correct information about COVID-19 infection and vaccines on its Q&A page [15,22] and by communicating information in various media. As misinformation and fake news vary from country to country and region to region, it is necessary to consider health communication tailored to Japanese methods.

Therefore, this study aimed to examine the association of COVID-19 prevention behavior with health literacy, COVID-19 knowledge, and fear of COVID-19 by age group.

The research question for this study was as follows: “Do factors associated with COVID-19 prevention behaviors differ by age?”

## Methods

### Study Design and Setting

A cross-sectional web-based survey was conducted from November 1 to November 5, 2021, among people aged 20-69 years. The participants were recruited online using Surveroid (Marketing Applications Inc). The primary analysis was a correlation analysis for each age group separated by 10 years. We used the Cohen medium effect size because we could not find any previous reference [23]. Thus, the effect size was set at *r*=0.3, with an alpha set at .05 and a beta set at .2. The required sample size was 84 participants per age group. Surveroid collected an extra 20% of the sample in case of response errors or omissions. Therefore, the final number of participants was calculated to be 102-103 per age group, for a total sample size of 512. Equal numbers of men and women were assigned to each age group. The study was described at the beginning of the web-based questionnaire, and completion of the questionnaire was considered as consent to participate in the study. The web-based questionnaires were collected in a randomized identification format without asking for personal information such as names or email addresses. The participants received a reward upon completion based on their registration status on Surveroid database. A survey request was sent to 8809 people via email. The survey was terminated when the target number of 512 participants was reached.

### Measures

The web-based questionnaire consisted of the following five factors or groups of items: (1) sociodemographic indicators and experiences during the COVID-19 pandemic, (2) COVID-19 prevention behaviors, (3) health literacy, (4) COVID-19 knowledge, and (5) fear of COVID-19. The questionnaire could be completed in 5 minutes.

#### Sociodemographic Indicators and Experiences During the COVID-19 Pandemic

The participants were asked about their sex, age, education level, experience with COVID-19 infection or close contact with an infected person, and the number of COVID-19 vaccine doses received.

#### COVID-19 Prevention Behaviors

The questionnaire was developed and used in previous studies based on the COVID-19 prevention behaviors presented by the Ministry of Health, Labour and Welfare [24]. This factor consisted of 9 items that measured outing or interpersonal contact and handwashing behaviors (Table 1). These items asked how much each action has been taken so far and were rated on a 5-point scale: 1=“not frequent at all,” 2=“not very frequent,” 3=“neither,” 4=“frequent,” and 5=“very frequent.” The total score is the sum of the scores of the 9 items, ranging from 9 to 45, with a higher score indicating more infection-preventing behaviors. The total score is the Behavioral Score (BS).

**Table 1:** COVID-19 prevention behaviors questionnaire.

Number	Item
1	Refrained from going out to places with large crowds of people.
2	Refrained from going out to enclosed places with poor ventilation.
3	Refrained from going out to places where you would be in close proximity to people, such as talking or vocalizing up close.
4	Stayed at home.
5	As soon as your work and errands were done, you went home.
6	You try not to meet people whenever you go out.
7	When meeting with people other than your roommate, you decided to keep a distance of at least two meters.
8	When you got home, you washed your hands for at least 20 seconds.
9	Frequent hand washing.

#### Health Literacy

Health literacy was assessed using Communicative and Critical Health Literacy (CCHL) [25]. The CCHL is a self-administered questionnaire developed by Ishikawa et al [25]. Its reliability and validity have been demonstrated in evaluating interactive and critical health literacy among the public. The CCHL consists of 5 items rated on a 5-point scale ranging from 1 (strongly disagree) to 5 (strongly agree). The mean score for all the questions was calculated. A higher mean score indicates higher ability. In a previous study conducted in Japan using the CCHL, the mean score of the CCHL was reported to be 3.58-3.7 [25,26].

#### COVID-19 Knowledge

The participants were asked whether the 6 information items on COVID-19 were correct or incorrect (Table 2). The questions were based on a selection of misinformation that was prevalent in Japan and was clearly listed as incorrect on the Q&A page related to COVID-19 provided by the Ministry of Health, Labour and Welfare [15,22]. The participants responded to the information using the 3 options, correct, unknown, or incorrect, and the total number of correct answers was counted as the correct answer score (CAS). Higher scores indicated a higher number of correct answers and greater knowledge of COVID-19, as presented in this study.

**Table 2:** COVID-19 knowledge questionnaire.

Number	Item	Answer
1	COVID-19 is vulnerable to heat, and low temperature water (25-35 degrees Celsius) has a bactericidal effect.	Incorrect
2	Alcohol disinfection is effective against COVID-19.	Correct
3	COVID-19 vaccine makes you infertile.	Incorrect
4	Vaccination can lead to infection with COVID-19.	Incorrect
5	The vaccine can be given during pregnancy, lactation, or while planning a pregnancy.	Correct
6	If a vaccinated person becomes infected with a mutated virus, he or she is likely to become seriously ill.	Incorrect

#### Fear of COVID-19

The fear of COVID-19 was assessed using the FCV-19S. The FCV-19S is reliable and valid for assessing COVID-19 fear in the general population [3]. The questionnaire was translated into Japanese and shown to be reliable and valid [16,27]. The FCV-19S is a 7-item self-administered scale. The minimum possible score for each question ranged from 1 (strongly disagree) to 5 (strongly agree). The total score was calculated by summing each item’s score, which ranged from 7 to 35. Higher scores indicate greater fear of contracting COVID-19. In April and August 2020, a previous study on FCV-19S in Japan reported mean scores of 21.25 and 16.67 [16,27].

### Statistical Analysis

The distribution of the studied variables was explored using descriptive analysis. The Kruskal-Wallis rank sum test was used to compare the scores of each item for each age group. If there was a significant difference in the Kruskal-Wallis rank sum test, the Tukey-Kramer test was added to determine which group had a significant difference. The relationship between BS, CCHL, FCV-19S, and CAS was reported using Spearman rank correlation analysis, as the assumptions of normality and homoscedasticity were violated for all indicators. In addition to the overall analysis, these analyses were performed separately for each age group to elucidate the differences. Next, multiple regression analysis was conducted for each age group, with BS as the dependent variable; CCHL, FCV-19S, and CAS as independent variables; and sex and age, which have been associated with health-related behaviors in previous studies, as adjustment variable [7,28]. When there was a correlation between the independent variables, multicollinearity (variance inflation factor [VIF]) was examined; if the VIF was <10, it was determined that there was no multicollinearity. The significance level for rejection of the null hypothesis was 5%. Statistical analysis was performed using the JMP Pro version 15.0 statistical software (JMP Statistical Discovery LLC).

### Ethics Approval

The Medical Ethics Committee of Kyoto University, Japan, approved this study (#R3215).

## Results

### Participants’ Characteristics

A total of 512 adults aged ≥20 years participated in the survey. The mean age was 44.3 (SD 14.3) years. All age groups were assigned approximately equal numbers across men and women. The distributions of educational level, COVID-19 infection or contact experience, and number of vaccinations are shown in Table 3.

**Table 3:** Participants’ characteristics.

Variable		Value					
		Total, n (%)	20-29 years, n (%)	30-39 years, n (%)	40-49 years, n (%)	50-59 years, n (%)	60-69 years, n (%)
Total		512 (100)	102 (19.9)	102 (19.9)	103 (20.1)	103 (20.1)	102 (19.9)
**Sex**							
	Man	257 (50.2)	51 (50.0)	51 (50.0)	52 (50.5)	52 (50.5)	51 (50.0)
	Woman	257 (49.8)	51 (50.0)	51 (50.0)	51 (49.5)	51 (49.5)	51 (50.0)
**Education level**							
	Middle school	10 (2.0)	1 (1.0)	3 (3.0)	1 (1.0)	2 (2.0)	3 (2.9)
	High school	164 (32.0)	30 (29.4)	34 (33.3)	28 (27.2)	34 (33.0)	38 (37.3)
	Technical school and junior college	120 (23.4)	22 (21.6)	22 (21.6)	26 (25.2)	26 (25.2)	24 (23.5)
	University	194 (37.9)	44 (43.1)	34 (33.3)	43 (41.8)	38 (36.9)	35 (34.3)
	Graduate school	24 (4.7)	5 (4.9)	9 (8.8)	5 (4.8)	3 (2.9)	2 (2.0)
**COVID-19 infection and close contact**							
	Both	7 (1.4)	3 (2.9)	1 (1.0)	3 (2.9)	0 (0)	0 (0)
	Only infection	6 (1.2)	2 (2.0)	3 (2.9)	1 (1.0)	0 (0)	0 (0)
	Only close contact	17 (3.3)	8 (7.8)	3 (2.9)	2 (1.9)	4 (3.9)	0 (0)
	Neither	482 (94.1)	89 (87.3)	95 (93.2)	97 (94.2)	99 (96.1)	102 (100)
**COVID-19 vaccine**							
	Double vaccination	392 (76.6)	67 (65.7)	79 (77.4)	76 (73.8)	79 (76.7)	91 (89.2)
	Single vaccination	33 (6.4)	10 (9.8)	6 (5.9)	7 (6.8)	7 (6.8)	3 (2.9)
	Unvaccinated	87 (17.0)	25 (24.5)	17 (16.7)	20 (19.4)	17 (16.5)	8 (7.9)

### Results of Correlation Analysis for Each Item

The results of the comparison of scores for each variable by age group are summarized in Table 4. The age group of 60-69 years had significantly higher BS than in 20-29 years. The age group of 60-69 years had a significantly higher CAS compared with the other age groups.

The results in Table 5 show that, for all participants, BS had significant positive correlations with CCHL, CAS, and FCV-19S; significant negative correlations with FCV-19S and CAS; and significant positive correlations with CCHL and CAS.

Analysis by age group shows that, in the 20-29 age group, there are significant positive correlations between BS, CCHL, and FCV-19S, and significant positive correlations between CCHL and CAS; in the 30-39 age group, there are significant positive correlations between BS, CCHL, and FCV-19S, and significant negative correlations between FCV-19S and CAS; in the 40-49 age group, there are significant negative correlations between BS and CCHL, and significant positive correlations between CAS and FCV-19S, and between FCV-19S and CCHL; in the 50-59 age group, BS and CAS have significant positive correlations, and CCHL and CAS have significant positive correlations; in the 60-69 age group, only CCHL and CAS have significant positive correlations.

**Table 4:** Comparison of each item by age group.

Variable	Value						
	Total, mean (SD)	20-29 years, mean (SD)	30-39 years, mean (SD)	40-49 years, mean (SD)	50-59 years, mean (SD)	60-69 years, mean (SD)	*P* value
BS^a^	35.11 (6.45)	33.86 (0.63)	34.55 (0.63)	35.51 (0.63)	35.12 (0.63)	36.50 (0.63^b^)	.02^c^
FCV-19S^d^	20.01 (5.62)	19.68 (0.56)	20.24 (0.56)	21.12 (0.55)	19.55 (0.55)	19.48 (0.56)	.32
CCHL^e^	3.55 (0.72)	3.47 (0.07)	3.44 (0.07)	3.54 (0.07)	3.65 (0.07)	3.66 (0.07)	.11
CAS^f^	3.58 (2.09)	3.15 (0.20)	3.31 (0.20)	3.11 (0.20)	3.71 (0.20)	4.62 (0.20^g^)	<.001^c^

^a^BS: behavioral score.

^b^The age group of 60-69 years had a significantly higher BS than in 20-29 years.

^c^*P*<.05.

^d^FCV-19S: Fear of COVID-19 Scale.

^e^CCHL: Communicative and Critical Health Literacy.

^f^CAS: correct answer score.

^g^The age group of 60-69 years had a significantly higher CAS than in the other age groups.

**Table 5:** The results of correlation analysis.

Variables	Compared variables	Values																
		Total			20-29 years			30-39 years			40-49 years			50-59 years			60-69 years	
		ρ	*P* value	ρ		*P* value	ρ		*P* value	ρ		*P* value	ρ		*P* value	ρ		*P* value
BS^a^	CCHL^b^	0.33	<.001^c^	0.42		<.001^c^	0.42		<.001^c^	0.46		<.001^c^	0.17		.09	0.10		.31
	CAS^d^	0.20	<.001^c^	0.17		.08	0.14		.16	0.25		.009^c^	0.24		.02^c^	0.14		.18
	FCV-19S^e^	0.22	<.001^c^	0.31		<.001^c^	0.22		.03^c^	0.28		.005^c^	0.17		.08	0.17		.08
FCV-19S	CCHL	0.06	.18	0.01		.89	0.10		.31	0.36		<.001^c^	–0.07		.49	–0.08		.42
	CAS	–0.16	<.001^c^	–0.09		.35	–0.25		.01^c^	–0.12		.24	–0.17		.095	–0.07		.47
CCHL	CAS	0.26	<.001^c^	0.36		<.001^c^	0.16		.11	0.14		.16	0.37		<.001^c^	0.20		.046^c^

^a^BS: behavioral score.

^b^CCHL: Communicative and Critical Health Literacy.

^c^*P*<.05.

^d^CAS: correct answer score.

^e^FCV-19S: Fear of COVID-19 Scale.

### Factors Associated With Prevention Behaviors

The results of the multiple regression analysis with BS as the dependent variable and sex and age as the adjustment variable are presented in Table 6. In all cases, a VIF of <10 was found. Thus, we determined that there was no influence on the multiple regression analysis. In the analysis of all age groups, all variables were significant, and BS was associated with CCHL, FCV-19S, and CAS. By age group, the following variables were significantly related to BS: CCHL and FCV-19S at 20-29 years; CCHL and FCV-19S at 30-39 years; CCHL, CAS, and FCV-19S at 40-49 years; and FCV-19S at 50-59 and 60-69 years.

**Table 6:** Multiple regression analysis with the behavioral score (BS) as the dependent variable and sex and age as adjustment variables.

Age group and analysis		β	95% CI	*P* value	
**Total**					<.001^a^
	CCHL^b^	.28	1.82 to 3.23		
	CAS^c^	.16	0.26 to 0.76		
	FCV-19S^d^	.29	0.25 to 0.42		
**20-29 years**					<.001^a^
	CCHL	.39	1.71 to 4.71	<.001 ^a^	
	CAS	.06	–0.36 to 0.76	.48	
	FCV-19S	.38	0.25 to 0.63	<.001^a^	
**30-39 years**					<.001^a^
	CCHL	.31	1.00 to 3.68	<.001^a^	
	CAS	.16	–0.05 to 1.03	.07	
	FCV-19S	.32	0.16 to 0.55	<.001^a^	
**40-49 years**					<.001^a^
	CCHL	.48	2.82 to 6.13	<.001^a^	
	CAS	.23	0.25 to 1.26	.004^a^	
	FCV-19S	.19	0.01 to 0.40	.04^a^	
**50-59 years**					.01^a^
	CCHL	.05	–1.37 to 2.33	.61	
	CAS	.19	–0.05 to 1.22	.07	
	FCV-19S	.21	0.02 to 0.47	.03^a^	
**60-69 years**					.004^a^
	CCHL	.18	–0.14 to 4.64	.07	
	CAS	.12	–0.23 to 1.12	.21	
	FCV-19S	.24	0.07 to 0.55	.01^a^	

^a^*P*<.05.

^b^CCHL: Communicative and Critical Health Literacy.

^c^CAS: correct answer score.

^d^FCV-19S: Fear of COVID-19 Scale.

## Discussion

### Principal Findings

This study aimed to examine the association between COVID-19 prevention behaviors and health literacy, COVID-19 knowledge, and fear of COVID-19 by age group. Overall, CCHL, CAS, and FCV-19S significantly influenced COVID-19 prevention behaviors. We also found a significant negative correlation between FCV-19S and CAS, and a significant positive correlation between CCHL and CAS. The correlations between FCV-19S and CAS, as well as between CCHL and CAS were determined to have little impact on the multiple regression analysis as the VIF was examined. However, multiple regression analysis by age group showed that the factors associated with COVID-19 prevention behaviors differed by age group. Among the 20-29, 30-39, and 40-49 years, multiple factors, including health literacy, influenced COVID-19 prevention behaviors. However, at 50-59 and 60-69 years, FCV-19S influenced COVID-19 prevention behaviors, whereas CCHL and CAS did not.

Regarding the characteristics of the participants in this study, the educational level in all age groups was higher than in the census results published in 2015 [29]. Therefore, the participants were considered to have a higher level of education than the average Japanese population. Regarding the experience of infection or close contact with patients with COVID-19, most participants across all age groups answered “neither.” The published infection rate of COVID-19 in Japan on January 1, 2022, was 1.4% [15], which was almost the same as the infection rate in this study. Most participants (76.56%) had received both vaccine doses. The difference in the number of vaccinations according to age is thought to be due to priority vaccination for older adults and people with chronic diseases [22]. In addition, the mean CCHL score of 3.55 for all participants is comparable to that of previous studies [25,26] and is at the same level of health literacy as the average Japanese. In this study, CCHL was lower than average for those aged 20-29 and 30-39 years, which is consistent with previous studies showing that health literacy is higher in older age groups [21].

In this paper, we first discuss the overall trends. The results of this study that health literacy, knowledge, and fear influence prevention behavior were similar to the previous studies. Health literacy was assessed using the CCHL, which was created to assess the public health literacy of the average citizen, and consists of higher-order communicative and critical health literacy rather than functional health literacy [25]. Communicative literacy is the ability to extract and understand information from a variety of communications and actively apply new information to changing circumstances in everyday activities [30]. Critical literacy refers to the ability to critically analyze and use information to better control a situation [30]. A previous study has shown that the higher the communicative or critical health literacy, the healthier the lifestyle [25]. This finding suggests that a high level of communicative or critical health literacy enables people to obtain information from various sources. Furthermore, it is likely that they can analyze the information obtained, leading to better behaviors. Primary studies have shown a relationship between health literacy and COVID-19 knowledge [13], and this study also showed a positive correlation between health literacy and COVID-19 knowledge. Therefore, even in the COVID-19 infodemic, people with high health literacy were able to obtain true information and adopt COVID-19 prevention behaviors without being misled by misinformation. These findings suggest that improving health literacy could help promote COVID-19 prevention behaviors. In addition, it is important to create an information dissemination system that allows people with low health literacy to obtain accurate information. Previous studies have shown that Japanese people have lower health literacy than Europeans [21]. This is because it is difficult to search for evidence-based information in Japan, as searches for diseases and symptoms often show information that is not available on official websites [21]. We believe that tools that enable anyone to access true information easily are needed.

Fear of COVID-19 was also associated with COVID-19 prevention behaviors. A previous study showed that the more threatening COVID-19 is perceived to be, the more prevention behaviors are taken [24]. In this study, as in the previous study, the greater the fear of COVID-19, the more prevention behaviors were adopted.

As mentioned above, health literacy, COVID-19 knowledge, and fear of COVID-19 are all relevant to prevention behaviors. However, panic and fear due to COVID-19 have been associated with depression and high stress states [31]. The results revealed a negative correlation between COVID-19 knowledge and fear of COVID-19. Therefore, promoting prevention behavior through correct knowledge of COVID-19, rather than fear of COVID-19, is considered more advisable from a citizen's mental health perspective. Communicating correct information, rather than fearmongering, is considered more important.

Next, we discuss the results according to age group. Among the 20-29 years, 30-39 years, and 40-49 years age groups, multiple factors, including health literacy, influenced COVID-19 prevention behavior. By contrast, in the 50-59– and 60-69–years age groups, only FCV-19S influenced COVID-19 prevention behaviors, whereas CCHL and CAS did not. One reason might be that the older age group had experienced influenza pandemics in 1957 and 1968 as well as the emerging infectious disease, severe acute respiratory syndrome pandemic in 2002 [32,33], and thus more easily adopted prevention behaviors, regardless of their health literacy and COVID-19 knowledge. In addition, in the COVID-19 pandemic, there is another possibility—COVID-19 has been reported to cause more severe symptoms at older ages [15] and is routinely reported on television and in newspapers in Japan. Therefore, older individuals are more likely to perceive the risk of infection as a threat. Furthermore, experts argue that older adults are vulnerable to anxiety and stress caused by the pandemic and are particularly fearful of social isolation and loneliness [34,35]. Against this background, fear of COVID-19 may have been a factor in prevention behaviors in the older age group, regardless of health literacy or COVID-19 knowledge. However, the relationship between fear and prevention behaviors is a vicious cycle that may lead to isolation due to prevention behavior that avoids seclusion and worsens mental health due to fear. We believe that prevention behaviors need to be supported by correct knowledge and health literacy and not solely based on fear. To this end, social support to reduce the stress caused by the pandemic, to protect against social isolation, and to provide correct knowledge is considered important.

Mature adults, similar to the overall trend, had multiple factors associated with prevention behaviors, including health literacy. One of the differences between younger and older adults is that social media use is higher among the younger age groups. Our contemporaneous study found that social media use was higher among those in their 20s and 30s than among the 50s and older age groups [36]. During the COVID-19 infodemics, high health literacy and correct knowledge may have been more important because a lot of misinformation was disseminated on SNS. Based on the results of this study, we believe that support tailored to age-specific characteristics is needed to promote prevention behaviors.

### Limitations

This study had several limitations. First, this was a cross-sectional study; therefore, causal relationships could not be established. Longitudinal studies must be conducted to confirm the causality. Second, the participants had a higher level of education than the general Japanese population. In addition, as this was a web-based survey, the participants were limited to internet users. Questionnaires should be administered to a wide range of individuals. Third, this study was conducted in November 2021, a period during which the number of COVID-19 cases in Japan had declined. The FCV-19S isolates in this study differed from those reported in previous studies [16,27]. The fear of COVID-19 may vary depending on the infection status and the type of virus prevalent. In addition, COVID-19 prevention behaviors may be altered by government policies. Therefore, further investigations of different infection statuses would be helpful. Fourth, this study collected questionnaires from participants only once. Therefore, the responses' reliability in this sample has not been verified. Therefore, we will need to examine the reliability of the responses in the future.

### Future Perspective

The COVID-19 pandemic is still ongoing, and we need to consider how to live with this virus. Governments and local authorities need to encourage people to take action to prevent the spread of infection and further lockdowns. The results of this study indicate that factors associated with prevention behavior differ by age, suggesting that there may be different means of promoting prevention behavior. However, information on COVID-19 is updated daily, making it difficult to generalize the results of this study. Therefore, it is necessary to expand the time period and geographical regions in future studies. In addition, longitudinal studies are needed to show causal relationships and to improve the level of evidence by providing health literacy education and interventions to alleviate fear.

### Conclusion

In this study, we identified the factors that influence COVID-19 prevention behaviors and found that these factors differed by age. In the age groups 20-29 years, 30-39 years, and 40-49 years, multiple factors, including health literacy, influenced COVID-19 prevention behaviors, whereas in the age groups of 50-59 and 60-69 years, only fear of COVID-19 influenced behavior. This suggests that tailored support according to age and risk factors is needed to promote prevention behaviors.

## Abbreviations

BS: behavioral score
CAS: correct answer score
CCHL: Communicative and Critical Health Literacy
FCV-19S: Fear of COVID-19 Scale
SNS: social network services
VIF: variance inflation factor

## Data Availability

The data supporting the findings of this study are available from the corresponding author upon reasonable request.
